# Beverage bottle capacity, packaging efficiency, and the potential for plastic waste reduction

**DOI:** 10.1038/s41598-021-82983-x

**Published:** 2021-02-25

**Authors:** R. Becerril-Arreola, R. E. Bucklin

**Affiliations:** 1 University of South Carolina, Columbia, USA; 2 University of California, Los Angeles, USA

**Keywords:** Environmental impact, Environmental impact, Sustainability

## Abstract

Plastic pollution is a pressing issue because authorities struggle to contain and process the enormous amount of waste produced. We study the potential for reducing plastic waste by examining the efficiency with which different polyethylene terephthalate (PET) bottles deliver beverages. We find that 80% of the variation in bottle weight is explained by bottle capacity, 16% by product category, and 1% by brand. Bottle weight is quadratic and convex function of capacity, which implies that medium capacity bottles are most efficient at delivering consumable product. Local data on PET bottle sales and municipal waste recovery validate the findings. A 20% shift in consumption from smaller to larger bottles could reduce the production of PET waste by over 10,000 t annually in the U.S. alone.

## Introduction

Waste from consumer goods packaging, including plastic, imposes downstream costs for disposal, recycling, and environmental damage^1–5^. Plastic waste is of particular importance because recycling and reuse rates are low and the material is durable, allowing mismanaged plastic waste to accumulate in the environment. Of all plastic packaging waste generated worldwide, 14% is recycled, 14% incinerated, 40% is landfilled, and 32% escapes collection^6^ (in the U.S., only 2% of plastic waste escapes collection^2^ and this favors the accuracy of our results.) Consumer products account for 70% of the entire market for plastic packaging^7^ and impose a cost to the environment, society, and economy that was estimated at 75 billion dollars per year in 2014 (notably, carbonated soft drinks packaged in plastic accounted for 9 billion)^4^. Disposed plastic packaging releases toxic solids that pollute water and soil, generate harmful emissions that pollute the air, and produce pervasive litter that threatens the lives and health of plants, animals, and humans^1–3,5,8^. Unless the problem is effectively addressed, these costs may surge in the future. A growing global population and a significant rise of per capita plastic consumption are predicted to double plastic waste generation over the next two decades, with the highest growth occurring in low-income countries (260%), upper-middle income countries (133%), and lower-middle income countries (133%)^8^.

Strategies for controlling plastic waste include both waste management practices (e.g., recycling, landfilling, converting plastic to energy) and innovation (e.g., using biodegradable plastics). All have shortcomings. Even as new technologies develop^9^, recycling is not always self-sustaining and landfilling and conversion to energy generate toxic pollutants. A significant reduction of plastic waste and its environmental consequences also requires source reduction^10,11^, defined by the U.S. Environmental Protection Agency as “any change in the design, manufacturing, purchase, or use of materials or products (including packaging) to reduce their amount or toxicity before they become municipal solid waste” ^11^*.* Waste professionals have long called for researchers and companies to find ways to reduce the plastic used^12^.

We explore the potential to reduce plastic waste by examining package efficiency variation across plastic beverage bottles sold to U.S. consumers. Here, efficiency is the quantity of consumable product delivered relative to the mass of plastic package used to contain it. Manufacturers and packaging companies can improve the efficiency of packaging by, for example, increasing the concentration of their products^13^, but some of these strategies are infeasible for many beverages (e.g., water) and “on-the-go” consumption occasions. The possibility of improving delivery efficiency by shifting the capacity of beverage bottles sold–with no change to the total amount of product delivered—has not yet received comparable attention.

We focus specifically on polyethylene terephthalate (PET), the most commonly used primary packaging material in non-alcoholic beverage categories^14,15^. (We do not consider secondary or tertiary packaging, such as trays and pallets used and disposed by retailers.) Because some state governments capture and report data on the PET waste stream, we can link local sales of consumer beverage containers to the plastic waste tonnages recorded. Also, among all plastic bottles, PET is the dominant packaging material, accounting for 62% in 2013^15^. [High-density polyethylene (HDPE) accounts for 36% but, because it is used for more diverse food and non-food product categories such as milk and detergent, the analysis of sales data for products packaged in HDPE would be far more complex and less reliable.] We also note that about 80% of PET produced is used to bottle non-alcoholic beverages^16^. Thus, most PET waste is associated with the non-alcoholic beverage industry and non-alcoholic beverages are most often packaged in PET. A focus on PET therefore provides the most tractable opportunity to study the problem with actual sales data because of the well-defined set of product categories involved. We follow industry, government^4^, and nonprofit^17^ studies and employ weight as our measure of PET usage. Because PET has a fixed density, weight also represents the quantity of material used.

## Results

The amount of PET required to deliver bottled beverages depends on numerous design and manufacturing factors (e.g., bottle capacity, the shape and texture associated with the brand, the oxidation rate of the contents, many parameters of the manufacturing technique). Because so many factors are involved, the mechanical design of plastic bottles is still, largely, a trial and error process^18^. No closed-form expression for the relationship between bottle weight and design attributes exists to our knowledge (most relevant academic studies rely on numerical methods). Thus, we opted for an empirical approach to quantify this relationship.

We collected data on PET container attributes for the product lines of a series of leading beverage brands that account for a large proportion of the U.S. market. This allowed us to model container weight as a function of container attributes, such as capacity, and calibrate these relationships. We then explored whether actual PET waste figures validate these findings. To do this, we modeled the reported tonnage of PET waste collected as a function of the local sales of PET beverage products and their bottle capacity mix. These two analyses shed light on the “costs” that less efficient package capacities potentially impose downstream.

In focusing on the U.S., we took advantage of complete and detailed retail sales data and a low proportion of PET escaping the waste management system^2,6^. (If we were to use data from jurisdictions where significant waste escapes the waste management stream, our estimates would lose accuracy.) Within the U.S., we focused on Minnesota because (i) its government reports PET waste collection figures reliably for most of the state’s counties, (ii) its patterns of non-alcoholic beverage consumption are close to the national average, and (iii) it collects a dominant share of PET (68%) from residential sources^19^ and residential waste is tightly connected to retail sales. Using retail sales data, we identified the beverage brands that dominated (in terms of market shares) the Minnesota market during years 2009–2013. We then collected and weighed all the bottles in their product lines. Following the definitions used in retailing, we group products into three major categories: carbonated drinks (including low calorie and carbonated soft drinks), juices and cocktails (including fruit drinks, fruit juices, vegetable juices, and cider), and non-carbonated water (including fruit-punch bases and syrups and non-refrigerated shakes, which constitute a negligible proportion of sales). These categories respectively account for 32%, 28%, and 40% of beverage ounces sold in the U.S.

### The determinants of bottle weight

We used the data collected from the bottles to estimate statistical models that explain bottle weight as a function of bottle capacity, product category, and brand (see “Methods” and Supplementary Materials). Bottle capacity is important because higher-capacity bottles require more plastic. Product category is important because thicker bottle walls are required to prevent the oxidation of more perishable beverages (such as juices) or to withstand the pressure of carbonation. In the sample (*n* = 187), the average weight of bottles is 27.56 g (95% CI, 19.72–35.39 g) for non-carbonated water, 27.38 g (95% CI, 25.59–29.16 g) for carbonated drinks, and 53.09 g (95% CI, 42.95–63.24 g) for juices and cocktails. Brand is important because it accounts for differences in styling, such as thicker walls for premium brands. The lowest average weight for a product line is 16.25 g (95% CI, 10.98–21.52 g) and the highest is 66.16 g (95% CI, 37.56–94.77 g).

After testing different functional specifications, we found that bottle capacity explains 80% of the variation in bottle weight, product category explains 16%, and brand 1% (see Table 1). In total, these account for 98% of the variation in bottle weight. Controlling for bottle capacity, a bottle of carbonated beverage requires on average 6.70 g (95% CI, 3.13–10.28 g) or 109% more PET than a bottle of non-carbonated water. A bottle of juice requires on average 21.60 g (95% CI, 17.80–25.40 g) or 351% more PET than a bottle of non-carbonated water. Controlling for both bottle capacity and product category, the product line with the heaviest bottles weighs on average 36.60 g (95% CI, 30.08–43.12 g) more than the product line with the lightest bottles.

**Table 1:** Ordinary least square estimates of the effect of bottle and product attributes on bottle weight.

Dependent variable	Bottle weight (g)				
	(1)	(2)	(3)	(4)	(5)
Bottle capacity (Oz)	0.832*** (0.032)	0.421*** (0.101)	0.797*** (0.276)	0.360*** (0.075)	0.330*** (0.067)
(Bottle capacity)^2^		0.004*** (0.001)	− 0.004 (0.005)	0.004*** (0.001)	0.004*** (0.001)
(Bottle capacity)^3^			0.00004 (0.00003)		
Carbonated drinks category				12.864*** (1.696)	
Juice and cocktails category				27.762*** (1.928)	
Non-carbonated water category				6.160*** (1.924)	
Intercept	6.738*** (1.386)	13.915*** (2.140)	9.704*** (3.585)		
Brand fixed effects	Not included	Not included	Not included	Not included	Included
Number of observations	187	187	187	187	187
*R*^2^	0.782	0.802	0.804	0.963	0.976
Adjusted *R*^2^	0.781	0.799	0.801	0.962	0.971
Columns compared by F-test		(2) vs (1)	(3) vs (2)	(4) vs (2)	(5) vs (2)
F-test D.F		1	1	2	27
F-test *p*		2.91 × 10^–5^	0.145	2.20 × 10^–16^	1.80 × 10^–6^
F-test statistic		18.360**	2.137	107.435***	3.283***

Numbered columns correspond to different model specifications. Numbers in parentheses are standard errors. F-tests compare model specifications.

*Pr ( >|t|) < 0.1; **Pr( >|t|) < 0.05; ***Pr( >|t|) < 0.01.

### The relationship between bottle capacity and bottle weight

**Figure 1:**
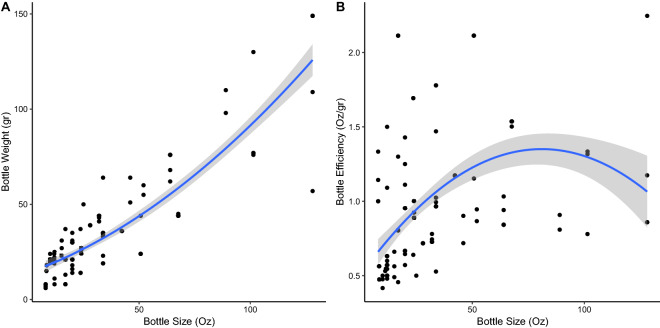
Relationships between bottle capacity and weight **(A)**, and between bottle capacity and efficiency **(B)** (*n* = 187). The blue line represents the best quadratic approximation and the gray envelope its standard error.

**Figure 2:**
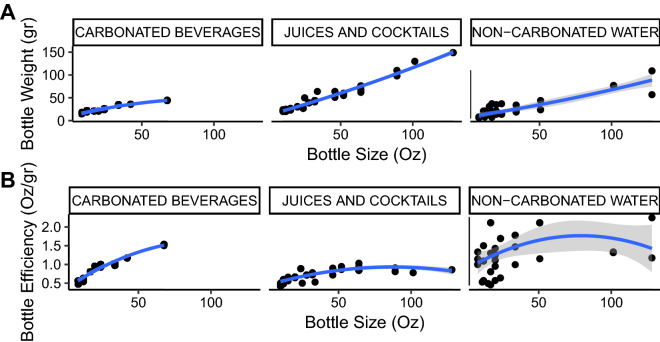
Relationships between bottle capacity and weight **(A)**, and between bottle capacity and efficiency **(B)** by beverage category (*n* = 187). The blue line represents the best quadratic approximation and the gray envelope its standard error.

Comparing models with different functional specifications for bottle capacity revealed that a quadratic model fits better than a linear model (F test, df = 1, *F* = 18.36, *p* < 0.01) but a cubic model does not fit better than the quadratic model (F test, df = 1, *F* = 2.14, *p* = 0.14). Bottle weight is thus a quadratic and *convex* function of bottle capacity and the efficiency of the bottle (its capacity to weight ratio) is a *concave* function of bottle capacity, as depicted in Fig. 1. The relationships exhibit the same shape across categories and the concavity is robust, as depicted in Fig. 2. (For carbonated drinks there are no bottles larger than 67.7 oz but the weight-efficiency curve is still concave). Thus, small bottles are the least efficient and large bottles actually may be less efficient than midsize bottles. In particular, the maximum efficiency is achieved at 76.60 oz. We discuss a possible theoretical explanation in Supplementary Text A.

### The relationship between bottle capacity and PET waste

We next use field data to assess the implications for plastic waste generation of the above relationship between bottle capacity and bottle weight. First, we obtained the PET tonnage collected for recycling in each Minnesota county for 2009 to 2013. Second, we procured county-level data on sales of beverage bottled in PET from The Nielsen Company (US), LLC (a company that collects and maintains retailing data from the grocery, convenience, drugstore, and mass merchandising channels^20^). Lastly, we gathered data from the U.S. Census Bureau for use as controls. We matched these three data sets by county and by year. This enables us to relate the capacities of the bottles actually sold to PET collection tonnage in the same year (beverages are typically consumed and their bottles disposed in just days or weeks after purchase).

**Figure 3:**
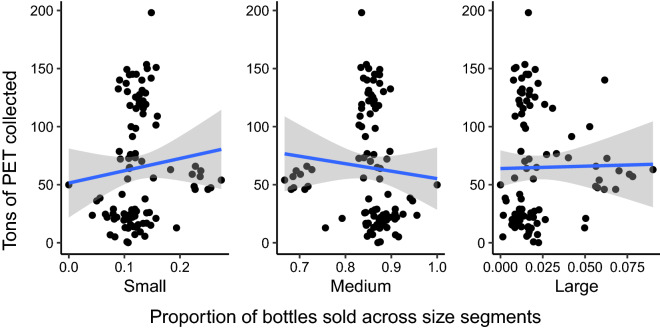
Relationships between PET collected for recycling and proportions of small, medium, and large bottles sold (*n* = 96). The blue line represents the best linear approximation and the gray envelope its standard error.

For model-free evidence and subsequent analyses, we categorized bottles into capacity groups. We followed beverage manufacturer standards^21^ and defined small bottles as those below 16 oz; these bottles target a specific segment of health-conscious consumers who typically consume them at home. Large bottles are those above 100 oz, targeted for family consumption. Midsize bottles fall between 16 and 100 oz, consumed “on-the-go” or at social occasions. We compared the proportion of bottles in each group sold in each county and year to the tonnage of PET collected in the same county and year, as presented in Fig. 3. The figure shows that PET tonnage is lower when the proportion of midsize bottles sold is relatively high. The figure also shows that many observations lie far from the lines of best fit. The patterns also are not statistically significant, suggesting the need for a multivariate model to control for other factors.

We next estimated a statistical model of PET waste tonnage as a function of the sales and capacities of PET beverage bottles sold. This model includes controls for time trends, the sales proportions for juices and carbonated drinks, and the sales of non-beverage products that could be packaged in PET (e.g., mouthwash, catsup, salad dressing, pickles, peanut butter). The model also includes county fixed effects to control for cross-county differences (e.g., differences in waste management systems and consumer behaviors). Specifications with additional control variables are reported in the Supplementary Materials. We separately measure the effects of small, midsize, and large bottles in the model. The results, presented in Column (1) of Table 2, confirm that small bottles increase the amount of PET collected for recycling more than midsize and large bottles do. The point estimate of the coefficient of small bottles is positive (*b* = 4.181, two-sided Student’s *t*-test, df = 61, *p* < 0.01) while the estimates for the other two capacities are negative (*b* = − 0.144, two-sided Student’s *t*-test, df = 61, *p* < 0.10 and *b* = − 0.084, two-sided Student’s *t*-test, df = 61, *p* < 0.01). Consistent with our results above, the point estimates indicate that midsize bottles produce less PET than large bottles. The two negative coefficients, however, are not statistically different and a model that combines the non-linear terms for midsize and large bottles [see Column (2)] exhibits similar fit. Thus, with respect to PET waste collected, the results show that large bottles are no more efficient than midsize bottles and may be slightly less efficient. We discuss the robustness of these analyses and potential alternative explanations in the “Methods” section.

**Table 2:** Feasible generalized least square estimates of the relationship between product category, bottle capacity, and PET waste collection

Dependent variable	PET collected (thousands of tons)	
	(1)	(2)
**Control terms**		
Year	− 177.069*** (65.281)	− 168.553** (79.828)
Non-beverage containers sold (thousands)	2.067*** (0.572)	2.195*** (0.690)
Containers of non-carbonated water sold (thousands)	− 1.773 (2.509)	− 0.257 (2.269)
Containers of juice and cocktails sold (thousands)	2.593** (1.044)	2.696** (1.163)
Containers of carbonated drinks sold (thousands)	0.101 (1.894)	0.172 (2.431)
**Term that accounts for a linear relationship**		
Beverage containers sold × container capacity (tons)	0.496 (0.386)	0.664 (0.513)
**Terms that account for nonlinearities**		
Beverage containers sold × container capacity^2^, small	4.181*** (1.181)	3.581*** (1.275)
Beverage containers sold × container capacity^2^, midsize	− 0.144* (0.084)	
Beverage containers sold × container capacity^2^, large	− 0.084*** (0.021)	
Beverage containers sold × container capacity^2^, midsize + large		− 0.073*** (0.019)
County fixed effects	Included	Included
Number of observations	96	96
*R*^2^	0.484	0.481
Adjusted *R*^2^	0.196	0.205

Numbered columns correspond to different model specifications. Numbers in parentheses are standard errors.

* Pr( >|t|) < 0.1; ** Pr ( >|t|) < 0.05; *** Pr ( >|t|) < 0.01.

To further explore the validity of these results, we compare reports of PET collected at the national level against the PET waste predicted by the bottle weight model based on sales data for the U.S. in 2013 (the last year in our data). We use the Nielsen database to compute the number of containers and ounces of beverage sold for each category-capacity combination in the U.S. as a whole. Then, we use the estimated bottle weight model to predict the weight of each bottle in the dataset and multiply it by the number of containers sold nationally to obtain the PET tonnage associated with each category-capacity combination at the national level. Adding across all category-capacity combinations, the model predicts that sales of non-alcoholic beverages at Nielsen-affiliated retailers would generate a total of 360.98 thousand tons of PET waste. As a comparison, in the U.S. overall, some 815.56 thousand tons of PET were collected for recycling in 2013^22^. However, Nielsen-affiliated retailers account for only a portion of U.S. sales volume (53% for supermarkets, 55% for drugstores, and 32% for mass merchandisers). The model’s prediction of only 360.98 thousand tons (or 44.3% of the 815.56 thousand tons) is therefore reasonable in light of the proportions of total sales by retailers that are covered by the Nielsen data.

### The potential benefits of shifting the capacity of bottles used

We performed a counterfactual exercise in which we simulate a 20% shift of sales of small beverages to midsize beverages while holding constant the number of ounces of beverage sold. For each product category and each bottle capacity below 16 oz, we reduce the number of ounces of beverage sold by 20% and compute the total reduction of beverage ounces sold. We then increase by this same amount the total number of ounces of product sold via medium capacity containers (between 16 and 100 oz) proportionally across product categories and bottle capacities. We then compute the counterfactual number of medium capacity containers needed in each category-capacity combination and use the bottle-weight model to compute the respective PET tonnage. Finally, we compare this predicted tonnage to the tonnage predicted with the original data and report the differences in Table 3. Through this procedure, we hold constant the total ounces of product sold within each category and account for geographic differences in consumption patterns across product categories and sizes (because we use data on actual consumption in all states). We find that a 20% transfer of beverage product sales from small to midsize containers reduces the PET used by over 1% in each year in the sample. Because in 2013 PET bottles in the U.S. generated 815.56 thousand tons of waste^22^, this makes the potential reduction equivalent to 9,978.45 tons of PET waste in that year. We also simulate shifts from large to midsize bottles and find that PET waste can be reduced by over 0.50%. Table 3 also suggests that the potential PET waste reduction associated with replacing small bottles has increased over time as small bottles have become more common in the U.S. In a global context, these growth estimates are likely to be conservative because plastic waste generation is predicted to grow much faster in low-income countries^8^.

**Table 3:** Projected PET waste reductions from shifting 20% of beverage consumption in small or large bottles to midsize bottles in the United States.

Year	20% shift from small to midsize		20% shift from large to midsize	
	% PET waste change	PET waste change (metric tons)	% PET waste change	PET waste change (metric tons)
2009	− 1.09	− 8915.85	− 0.60	− 4903.28
2010	− 1.08	− 8805.14	− 0.56	− 4565.96
2011	− 1.12	− 9137.95	− 0.54	− 4372.67
2012	− 1.19	− 9725.23	− 0.51	− 4190.36
2013	− 1.22	− 9978.45	− 0.50	− 4058.69
2014	− 1.23	− 10,044.33	− 0.50	− 4061.85
2015	− 1.29	− 10,544.72	− 0.47	− 3843.51

Projections are based on actual beverage sales figures at the national level and hold constant the total number of ounces of beverage sold in each product category.

## Discussion

We used a sample of beverage bottles to study bottle efficiency. The myriad factors that determine bottle weight prevent the development of an exact mathematical model to predict bottle weight and therefore existing work on the topic is scant, empirical, and largely applied. Furthermore, previous findings are mixed. Gell^23^ measured the capacity and weight of a sample of packaged good containers; the findings indicated that larger containers were more efficient in some categories but not others. The ULS Report^17^ found that larger containers—also sampled across a variety of grocery categories and packaging materials—were as or more efficient than smaller ones. Neither study reported findings for the beverage categories we examine (carbonated drinks, juices and cocktails, and non-carbonated water) nor did either study focus on PET. To the best of our knowledge, no study has yet examined whether manufacturers’ selections of container attributes, such as capacity, and their corresponding sales are reflected in actual waste stream data.

**Figure 4:**
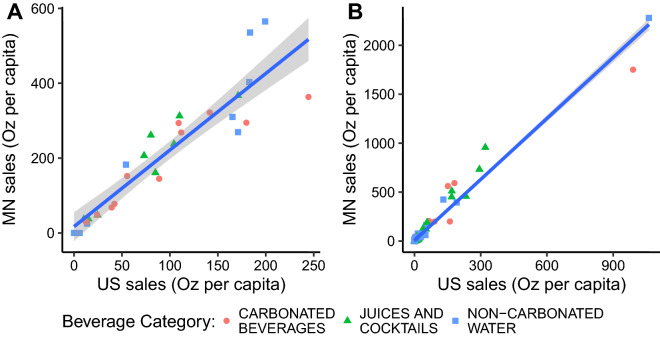
Sales per capita in Minnesota and the entire U.S. for all focal brands (**A**, *n* = 30) and for all size-category combinations available in Minnesota (**B**, *n* = 204). The blue line represents the best linear approximation and the gray envelope its standard error.

Using our sample and empirical model, we find small containers to be clearly less efficient than midsize and large ones. We find further support for this result by replicating the results with beverage sales data and PET waste data reported at the county level in Minnesota. The results of the first and more fundamental analysis can however be extrapolated to the national level because the mix of beverage sales across brands and sizes in Minnesota is similar to the mix in the U.S. as a whole. Figure 4 shows that the different brands and different bottle capacities considered in the first analysis exhibit similar sales patterns at the Minnesota and national levels. We thus expect the estimated bottle weight model to hold at the national level, even if consumption per capita of bottled beverages in Minnesota is different from the national average (we account for these differences in our counterfactual scenarios).

Our findings may be translated into actual PET waste reduction through different, non-mutually-exclusive strategies. Such strategies may focus on shaping consumer preferences and allow demand to influence the behavior of manufacturers, bottlers, and retailers. If demand for small containers decreases, both sales volumes and markups of small beverages may decrease and manufacturers and retailers may profit from retiring the smallest sizes. Shorter product lines are less expensive to produce and distribute^24^ and brands perceived as environmentally friendly can reach higher sales and markups^25,26^. The feasibility of demand-driven changes like the one proposed is evidenced by the growing consumer interest in refillable containers^25^ and the ensuing response of manufacturers to introduce refillable containers for products ranging from orange juice to deodorant^27^.

Consumers choose small and large beverages for consumption at home or at social occasions and medium sizes for consumption away from home. In addition, choices are heavily influenced by affordability, health, and environmental concerns^25^. Price-sensitive consumers prefer large beverages because price per ounce is generally lower for large formats. Substitution towards midsize beverages may necessitate consumption taxes on large, single-use containers. In contrast, health-oriented consumers often substitute large beverages with small beverages at home because the latter facilitate the consumption of small portions. Midsize beverages could meet the need for smaller portions if, for example, their bottles featured printed scales to help in fractioning their contents. Finally, environmentally-concerned consumers prefer beverages bottled in recycled materials, plant-based plastics, and other environmentally-friendly options^25^ but appear unaware of differences in efficiency across container sizes. These consumers may be encouraged to switch to medium capacity bottles if bottle efficiency was reported on the label. Increased awareness of efficiency differences may suffice. Surveys suggest that about 60% of consumers support reporting product sustainability on shelf tags and packaging. About 36% of consumers support taxes on single-use plastic containers^25^.

A potential concern with shifting beverage sales from small to midsize containers is the possibility that consumption might increase, leading to adverse health consequences^28,29^. While this may not be a concern with non-carbonated water, it could be for the other beverage categories. We also recognize that, in the case of carbonated drinks, such shifts towards midsize bottles may require additional measures because carbonation is quickly lost after the first opening of the container. In addition, small containers may reduce the repeat use of containers and thus exposure to potentially-infectious microbial growth^30^. These are important topics for future research.

## Materials and methods

### Models

This section presents our empirical models. We first model the relationship between bottle weight and bottle capacity for PET beverage containers. Next, we model PET waste tonnage as a function of beverage sales and the capacity mix of bottles sold.

First, we model bottle weight as a function of product category, bottle capacity, and brand. We consider bottle weight to be a general function of bottle capacity but, for tractability, we use a second order Taylor-series approximation such that

1 $${BOTWGT}_{i}={\alpha }_{k(i)}+{\delta }_{b(i)}+\beta {BOTCAP}_{i}+\gamma {BOTCAP}_{i}^{2}+{\epsilon }_{i},$$

where ${BOTWGT}_{i}$ is bottle weight (in grams) of the bottle of product *i*, ${\alpha }_{k(i)}$ is a fixed effect for the product category *k* of product *i*, ${\delta }_{b(i)}$ is a fixed effect for the brand *b* of product *i*, ${BOTCAP}_{i}$ is bottle capacity (in ounces), and ${\epsilon }_{i}$ represents the higher order terms of the Taylor series as well as other unobserved design and manufacturing factors. We estimate this model using ordinary least squares (OLS). The results are reported in Table 1. Because the brand fixed effects nest the category fixed effects, we do not include both sets of fixed effects simultaneously.

Next, we assess the relationship between PET waste and beverage sales. Because brand explains only about 1% of the variation of the bottle weight, we assume ${\delta }_{b(i)}=0$, ∀ *i*. The PET *generated* by the sales of product *i* in county *c* and year *t*, ${BOTPET}_{ict}$, is given by ${BOTPET}_{ict}={BOTQTY}_{ict} {BOTWGT}_{i}$, where ${BOTQTY}_{ict}$ is the number of bottles of product *i* sold. Combining this with Eq. (1) and aggregating across all *N* available products, we can write the total PET waste of beverage products *i* = 1…*N* sold in county *c* in year *t*, ${BOTPET}_{ct}$, as follows:

2 $${BOTPET}_{ct}=\sum_{i=1}^{N}{BOTPET}_{ict}=\sum_{i=1}^{N}{BOTQTY}_{ict} {BOTWGT}_{i}=\sum_{i=1}^{N}\left({\alpha }_{k(i)}{BOTQTY}_{ict}+\beta {BOTQTY}_{ict}{BOTCAP}_{i}+\gamma {BOTQTY}_{ict}{BOTCAP}_{i}^{2}+{\stackrel{\sim }{\epsilon }}_{ict}\right),$$

where ${\stackrel{\sim }{\epsilon }}_{ict}={BOTQTY}_{ict}{\epsilon }_{ict}$ is proportional to ${BOTQTY}_{ct}$ and thus heteroscedastic by construction.

Recognizing that up to 20% of PET waste is associated with sources other than non-alcoholic beverages, we define the amount of PET collected at the county-yearly level as

3 $${PET}_{ct}={\tau }_{c}+\phi t+\delta {NONBEVBOTQTY}_{ct}+\sum_{i=1}^{N}{\alpha }_{k(i)}{BOTQTY}_{ict}+\beta\sum_{i=1}^{N}{BOTQTY}_{ict}{BOTCAP}_{i}+\gamma \sum_{i=1}^{N}{BOTQTY}_{ict}{BOTCAP}_{i}^{2}+{\stackrel{\sim }{\epsilon }}_{ct},$$

4 $$={\tau }_{c}+\phi t+\delta {NONBEVBOTQTY}_{ct}+\sum_{i=1}^{N}{\alpha }_{k(i)}{BOTQTY}_{ict} + \beta\sum_{i=1}^{N}{BEVQTY}_{ict}+\gamma \sum_{i=1}^{N}{BEVQTYBOTCAP}_{ict}+{\stackrel{\sim }{\epsilon }}_{ct},$$

where we implicitly define ${\stackrel{\sim }{\epsilon }}_{ct}=\sum_{i=1}^{N}{\stackrel{\sim }{\epsilon }}_{ict}$, ${BEVQTY}_{ict}={BOTQTY}_{ict}{BOTCAP}_{i}$, ${BEVQTYBOTCAP}_{ict}={BEVQTY}_{ict}{BOTCAP}_{i}$, ${NONBEVBOTQTY}_{ct}$ is the number of containers of non-beverage products sold in county *c* and year *t*, ${\tau }_{c}$ are county-specific fixed effects that account for differences in collection procedures and recycling practices across counties, and the term *φt* accounts for unobserved trends.

We note that Model (4) is hard to interpret. Depending on the values of the variables, a positive value of the parameter $\gamma$ could represent a convex relationship between waste and average bottle capacity, but could also represent an increase in PET waste when small bottles are uncommon. We therefore discretize the model, starting from Eq. (2) to obtain:

$${BOTPET}_{ct}=\sum_{i=1}^{N}\left({\alpha }_{k(i)}{BOTQTY}_{ict}+\beta {BEVQTY}_{ict}+\gamma {BEVQTY}_{ict}{BOTCAP}_{i}+{\stackrel{\sim }{\epsilon }}_{ict}\right)$$

$$=\sum_{k=1}^{3}{\alpha }_{k}\sum_{i\in {\Omega }_{k}}{BOTQTY}_{ict}+\beta \sum_{i=1}^{N}{BEVQTY}_{ict}+\sum_{i=1}^{N}{\gamma BEVQTY}_{ict}{BOTCAP}_{i}+{\stackrel{\sim }{\epsilon }}_{ct}$$

$$=\sum_{k=1}^{3}{\alpha }_{k}{BOTQTY}_{kct}+\beta {BEVQTY}_{ct}+\sum_{g=1}^{3}{\gamma }_{g}{BEVQTYBOTCAP}_{gct}+{\stackrel{\sim }{\epsilon }}_{ct},$$

where ${\Omega }_{k}$ represents the subset of products that correspond to category *k*, ${BOTQTY}_{kct}=\sum_{i\in {\Omega }_{k}}{BOTQTY}_{ict}$ is the number, in thousands, of containers of product category *k* = 1,…,3 sold in county *c* in year *t,* ${BEVQTY}_{ct}=\sum_{i=1}^{N}{BEVQTY}_{ict}$ is the sales of beverage (in tons), *G*_*g*_ is the set of all products *i* whose capacity places them into capacity group *g,* and ${BEVQTYBOTCAP}_{gct}=\sum_{i\in {G}_{g}}{BEVQTY}_{ict}{BOTCAP}_{i}$. Then, accounting for non-beverage containers, we write the total amount of PET collected for recycling as

5 $${PET}_{ct}={\tau }_{c}+ \phi t+\delta {NONBEVBOTQTY}_{ct}+\sum_{k=1}^{3}{\alpha }_{k}{BOTQTY}_{kct}+\beta {BEVQTY}_{ct}+\sum_{g=1}^{3}{\gamma }_{g}{BEVQTYBOTCAP}_{gct}+{\stackrel{\sim }{\epsilon }}_{ct},$$

which is equivalent to the model specification for the estimates appearing in Table 2, Column (1). We estimate Model (5) and similar models reported in the Supplementary Text using linear panel techniques and Generalized Least Squares (see Estimation below).

### Data

We assembled our data from multiple sources, including a non-probabilistic sample of bottles acquired from retailer stores, the Minnesota Pollution Control Agency (MPCA), Nielsen, and the U.S. Census Bureau. We next describe the key sources and variables.

#### Bottle data

Using the Nielsen data, we computed the total ounce sales of beverage of each product line sold in Minnesota between 2009 and 2013. We sort the sales and identify 51 leading brands that represent 62% of market sales in beverage ounces. The remainder of the market consists of 1462 lines with an average market share of 0.02% and therefore these individually have little effect on the results. Following a standard procedure, we conducted an extensive search across retailers and states to purchase all bottles in each of the selected product lines. Once purchased, we cleaned and dried the bottles, removing labels, caps, cap rings, other non-PET components, and any remaining label glue. The final sample consisted of 187 bottles.

#### Waste data

Our data on collected PET is from the MPCA, to whom waste and recycling haulers must report annually to operate in Minnesota. The data span different counties as well as materials (e.g., glass, mixed paper, and PET) for the 87 counties in Minnesota during the years 2009 to 2013 (other years were available but had to be discarded because of changes in the reporting procedures). While several states report waste collection annually for different counties, we found no other state that consistently reports PET waste at the county level. Data we examined from other states exhibited patterns likely to reflect changes in reporting procedures and we therefore have focused on the Minnesota data. We define the variable ${PET}_{ct}$ as the total tonnage of PET collected for recycling from residential sources in county *c* in year *t*. Data on mixed waste (including foods, plastics, metals, etc.), ${WASTE}_{ct}$, is also available for a slightly smaller number of counties and the same years.

#### Nielsen store scanner data

To obtain the sales of different bottle capacities at the county level, we use store-level data from Nielsen. We focus on the three product categories that rely on PET for packaging: soft drinks-non-carbonated (including bottled water and teas), carbonated beverages (including soda), and juices and cocktail mixes. Nielsen reports the quantity of beverage associated with each Universal Product Code (UPC) in ounces and we identified 156 different bottle capacities. Nielsen also reports the container type and broad material type (e.g., plastic and aluminum can) for most UPCs. For UPCs without packaging material information, we imputed it using other years or inferred it from pictures in UPC databases and search engines. We excluded UPCs for beverages not packaged in plastic bottles (e.g., beverage powders distributed in envelopes or canisters and liquid beverages distributed in cans, cartons, and aseptic pouches).

We aggregate within counties and years to compute the variables ${BEVQTY}_{ct}$ (the tons of beverages sold), ${BOTQTY}_{ct}$ and ${BOTQTY}_{kct} k=1,...,3$ (the number of beverage bottles sold), and ${WPROPBOT}_{gct}$, *g* = 1,…,3 (the proportion of bottles in capacity group *g* sold). To account for sources of PET outside of the non-alcoholic beverage categories, we collected data for 941 additional, non-beverage product categories in the following departments: dry grocery, non-food grocery, frozen foods, health and beauty, and general merchandise. We use their sales data to compute the number of non-beverage bottles sold, ${NONBEVBOTQTY}_{ct}$.

Nielsen data are available for 67 counties for the MPCA dataset period. We exclude Ramsey county because of unusually low sales for its demographics (a dominant retailer in the area may not be included in the data). We also exclude observations with zero PET collection, as that is more likely to be due to lack of collection rather than lack of PET use. The final sample consists of 26 counties and up to 5 years per county, giving a total of 96 observations (not all years are available for all counties).

#### U.S. Census Bureau

We draw from the American Community Survey to construct the following county-year control variables for robustness analyses: population density, average income, unemployment, median age, prevalence of bachelor degrees, proportion black and Hispanic, proportion of families with children, and average household size. Details on these variables are in Supplementary Text B. Summary statistics are in Supplementary Text C, Tables S1 and S2.

### Estimation

We estimate the models predicting bottle weight using ordinary least squares (OLS). To estimate the models predicting PET collected, we use feasible generalized least squares (FGLS) with errors clustered at the county level. The FGLS procedure accounts for potential heteroscedasticity that might arise from differences in population size and economies across counties and from the construction of the residuals. We also accounted for serial correlation but found that the results were not significantly different. (This may be because either serial correlation is not present or the small size of the dataset and the limited number of years for each county is insufficient to recover serial correlation.)

In addition, the explanatory variables in the PET model are highly correlated by construction. This could threaten the stability of the results. To address this, we remove the common variance of highly correlated explanatory variables through regression^31^. In particular, we regress the explanatory variables on each other sequentially to reduce their correlations. To assess whether this procedure may affect the results, we also estimate the models with the original data. The results are unaffected for the discretized model. Collinearity is too severe to yield stable results without the correction procedure for the continuous model.

### Tests of robustness and alternative explanations

In modeling PET waste as a function of the bottle capacity mix, issues could arise with respect to identification. We test for the possibility that the results may be due to possible correlation between the predictor variables and omitted variables. In particular, we assess the possibility that differences in garbage sorting among consumers of different counties or over time may drive the results. We extend the full specification by including demographic variables known to correlate with the garbage sorting behaviors of consumers^32^. The results are consistent with our findings and we report them in Supplementary Text D, Table S3. Finally, we discuss how our approach addresses potential simultaneity, measurement error, and other omitted variables.

#### Simultaneity

Biases in our estimates could arise if consumers choose product sizes (and hence bottle capacities) in response to the amounts of plastic waste they generate. While consumers may limit the quantity of products they consume to reduce waste, they are seldom aware of the relationship between product sizes and waste (as suggested by previous studies^33^). When considering the environmental friendliness of beverages, consumers most often consider the bio-degradability of the bottle and the bottle’s material source (e.g., recycled plastic and plant-based plastic) but not bottle capacity (which is most often determined by the consumption occasion^34^). In a survey conducted by the market research company Mintel, minimization of unnecessary packaging material was only the 9th most important attribute for consumers choosing bottled beverages^35^. Furthermore, container (as opposed to product) sustainability has a small correlation with purchase intent (and thus possibly no correlation with actual behavior), probably because the most environmentally friendly behavior is not to buy bottled beverages at all^34^.

#### Measurement errors

Measurement error of the dependent variable could arise if the rate of consumer recycling differs across counties and/or over time. We examined the recycling policies of a sample of Minnesota counties and found them to be quite similar. Consumers receive additional recycling bins at no cost, but must pay for additional waste bins. Hence, incentives are in place to encourage residential recycling and minimize the non-recyclable waste stream. The data provided by the MPCA show that, for 2013, the PET found in mixed municipal waste (i.e., collected garbage) is less than 0.80 percent of all PET collected (either as mixed municipal waste or recycling). Thus, there is relatively little PET that escapes recycling collection in Minnesota. Similarly, only about 2% of total plastic waste was mismanaged (littered or dumped) in the U.S. ^6^. We also searched for but found no change in collection policies during the period of study. Nevertheless, we control for remaining potential differences in recycling rates across counties and over time by including county-fixed effects and a time trend. We also account for heteroscedasticity that measurement errors might induce in the residuals.

Another potential concern is that consumers may recycle bottles of certain capacities more so than others; if so, this effect could confound the relationship between the capacity of bottles sold and the generation of PET waste. Previous studies suggest this is unlikely to occur. For example, Trudel and Argo^33^ studied how product size and changes in product size and shape during consumption affect the decision to recycle paper and beverage cans. They found that it is the change in product size and shape, not the product’s actual size, which affects the decision to recycle. To further address this, we extend our model to include demographic variables known to influence recycling rates^32^ (see also our robustness tests in Supplementary Text D, Table S3).

#### Correlated omitted variables

If the assortment of PET bottle capacities in beverages is similar to those for other products also packaged in PET, the effect of assortment mix in the beverage categories could be overstated. Because bottled beverages are the main source of disposed PET (about 80% worldwide^7^), we believe that this is unlikely to induce appreciable bias. Nevertheless, we control for the sales of containers in 941 other grocery categories.

We also note that Nielsen stores account for slightly more than half of the all commodity volume of Minnesota’s retailers. Because store-level sales data from non-Nielsen reporting retailers are not available, we are unable to control for their assortments. Thus, we implicitly assume that the Nielsen stores’ assortments are representative of the local market at the county level.

## Supplementary Information

Supplementary Information.

Supplementary Dataset.

## Data Availability

Bottle data are available from the authors. Waste collection data are available from the Minnesota Pollution Control Agency (MPCA, https://www.pca.state.mn.us/). Nielsen data are available from the University of Chicago (https://www.chicagobooth.edu/research/kilts/datasets/nielsen).
