# Supplementary material for: Beverage bottle capacity, packaging efficiency, and the potential for plastic waste reduction
Source: Sci Rep. 2021 Feb 25;11:3542. doi: 10.1038/s41598-021-82983-x (PMC7907389; doi:10.1038/s41598-021-82983-x)
Supplement: Supplementary file 1 — Supplementary Information. [file 41598_2021_82983_MOESM1_ESM.docx]

**SUPPLEMENTARY MATERIALS**

**Beverage Bottle Capacity, Packaging Efficiency, and the Potential for Plastic Waste Reduction**

**Authors:**

R. Becerril-Arreola^1^*, R. E. Bucklin^2^.

**Affiliations:**

^1^University of South Carolina.

^2^University of California, Los Angeles.

*Correspondence to: [rafael.becerril@moore.sc.edu](mailto:rafael.becerril@moore.sc.edu).

A. The Relationship between Bottle Weight and Bottle Capacity

Define the efficiency of a container as the ratio of the weight of the product to the weight of the container. To compute the efficiency of a bottle as a function of capacity, we need to compute the weight of the bottle as a function of volume. For a cylindrical container (an approximation of the bottle), the weight of a bottle is $W(V)=\rho S\left( V \right)T\left( V \right)$, the product of the material’s density *ρ*, the surface of the bottle *S*, and its average thickness *T*.

The surface and volume of the cylindrical bottle are given by $S=2\pi r\left( r+h \right)$ and $V=\pi r^{2}h$, respectively, where *r* is the radius and *h* is the height of the cylinder. It follows that, for a constant radius to height ratio, the relationship between bottle capacity and surface is$S(V)=K_{1}V^{2/3}$. This is increasing but concave because the rate at which surface increases with capacity is decreasing. This suggests that large bottles could be more efficient.

Given a desired degree of bottle strength, bottle wall thickness must increase with bottle capacity *(36)*. Following past work *(37)*, average wall thickness can be described as a quadratic function of bottle radius; that is $T(V)=a_{1}+b_{1}r+c_{1}r^{2}$ for some constants *a*_1_, *b*_1_, and *c*_1_. Because, for a constant height to radius ratio, bottle radius satisfies $r\left( V \right)\propto V^{1/3}$, one can write wall thickness as $T(V)=a_{2}+b_{2}V^{1/3}+c_{2}V^{2/3}$ for some constants *a*_2_, *b*_2_, and *c*_2_. After grouping and defining new constants *a*, *b*, and *c*, one can write

$W\left( V \right)=aV^{2/3}+bV+cV^{4/3}+d$,

where the term *d* is added to account for the weight of the bottle’s neck.

Even if both the relationship between surface and capacity and the relationship between wall thickness and capacity are concave, theory cannot guarantee the concavity of the functional relationship between bottle weight and capacity. The second derivative of $W\left( V \right)$ is

$$\frac{d^{2}W\left( V \right)}{dV^{2}}=-\frac{2}{9}aV^{-4/3}+\frac{4}{9}cV^{-2/3}$$

which could be positive or negative depending on the values of *a*, *c*, and *V*. Hence, depending on the range of capacities and thickness under consideration, weight could be a linear, convex or concave function of capacity. We therefore evaluate the relationship empirically.

Some industry studies suggest that container weight is a mildly concave function of container capacity for a number of product categories *(23,17)* but convex for others *(17)*. However, for small bottles of water, the relationship has also been found to be linear *(38)*. One important reason for these differences is that different products require different wall thickness for different container capacities. For example, in the case of carbonated drinks, thick walls are required to contain the pressure created by the carbon dioxide *(39)*. At the same time, the pressure helps maintain the shape of large bottles so that, for carbonated drinks, the thickness of the bottle walls need not increase as fast as juices and bottled water. Juices also require thicker walls to reduce permeability and retard the decomposition of the juice. In sum, to understand the relationship between bottle weight and capacity, we rely upon an empirical analysis of bottles available from leading beverage product lines.

B. Variable definitions

Variables derived from Minnesota’s Pollution Control Agency and related constructs

- ${PET}_{ct}$ is the total tonnage of PET collected for recycling from residential sources in county *c* and year *t*.
- *BOTPET_ict_* is the total tonnage of PET from product *i* sold in county *c* during year *t* (used in model derivation, not in dataset).
- *BOTPET_ct_* is the total tonnage of PET used in beverage containers in county *c* during year *t* (used in model derivation, not in dataset).
- *WASTE_ct_* is the total tonnage of mixed waste (including foods, plastics, metals, etc. but excluding materials for recycling) collected in county *c* and year *t*.

Variables derived from Nielsen data:

- ${AVBOTCAP}_{g}$ is the average capacity of bottles in group *g* across all counties and years
- *BEVQTY_ct_* represents the total sales of beverages, in tons, in county *c* and year *t*
- ${BEVQTYBOTCAP}_{ct}=\sum_{i=1}^{N} {BEVQTY}_{ict}{BOTCAP}_{i}$
- *BOTCAP_i_* represents the capacity of the bottle of product *i*, measured in ounces of liquid it can carry
- *BOTQTY_ict_* represents the total number of bottles of product *i* sold in county *c* and year *t*
- *BOTQTY_ct_* represents the total number of bottles of all products sold in county *c* and year *t*
- *BOTWGT_i_* represents the weight of the bottle of product *i* (only the PET component included), measured in grams
- ${NONBEVCTNRCAP}_{ct}$ the capacity of containers of non-beverage products sold
- ${NONBEVPRODQTY}_{ct}$ the number of tons of non-beverage product sold
- ${NONBEVBOTQTY}_{ct}$ the number of non-beverage containers sold
- *PROPQTY_gct_* represents the proportion of tons of beverage delivered in bottles of capacity group *g*
- ${BEVQTYBOTCAP}_{gct}$ is the weighted proportion of bottle sold, and equals the product ${BEVQTY}_{ct}{AVBSIZE}_{g}{PROPQTY}_{gct}$

Variables provided by the U.S. Census Bureau:

- *AVHHSIZE_ct_* is the average size of households in county *c* and year *t*
- *AVINCOME_ct_* is the average income of households in county *c* and year *t*
- *MEDIANAGE_ct_* is the median age of inhabitants of county *c* in year *t*
- *PERCBACHELOR_ct_* is the percentage of individuals with bachelor degree or higher among the population in county *c* and year *t*
- *POPDENS_ct_* is the population density in county *c* and year *t*
- *PROPCHILDREN_ct_* is the proportion of families with children in county *c* and year *t*
- *PROPPOPBLACK_ct_* is the proportion of black individuals in county *c* and year *t*
- *PROPPOPHISP_ct_* is the proportion of Hispanic individuals in county *c* and year *t*
- *UNEMPRATE_ct_* is the unemployment rate in county *c* and year *t*

C. Descriptive statistics

Table S1 presents summary statistics of variables for analysis of county-waste data. Table S2 presents correlations among the transformed variables.

D. Full results and robustness tests for PET model

Table S3 presents results of additional robustness tests for the discretized model of county-waste data.

**Table S1. Summary statistics of main variables (original data).**

| Statistic | Mean | St. Dev. | Min | 25^th^ Pctl | Median | 75^th^ Pctl | Max |
| --- | --- | --- | --- | --- | --- | --- | --- |
| $PET$ | 2,052.199 | 5,925.299 | 0.036 | 58.389 | 295.393 | 1,084.130 | 40,590.060 |
| $NONBEVBOTQTY$ | 19,247.120 | 52,429.120 | 1.065 | 589.742 | 3,587.952 | 10,510.400 | 349,809.100 |
| ${BOTQTY}_{water}$ | 1,139.462 | 3,484.454 | 0.003 | 29.089 | 104.977 | 429.479 | 23,719.090 |
| ${BOTQTY}_{juice}$ | 549.288 | 1,514.140 | 0.009 | 15.536 | 69.490 | 319.227 | 10,685.580 |
| ${BOTQTY}_{soda}$ | 775.814 | 2,070.651 | 0.029 | 25.941 | 121.976 | 494.264 | 14,307.270 |
| $BOTQTY$ | 549.288 | 1,514.140 | 0.009 | 15.536 | 69.490 | 319.227 | 10,685.580 |
| $BEVQTY$ | 2,052.199 | 5,925.299 | 0.036 | 58.389 | 295.393 | 1,084.130 | 40,590.060 |
| $BEVQTYBOTCAP$ | 112,078.400 | 346,128.200 | 1.292 | 2,565.048 | 12,236.100 | 48,433.190 | 2,388,665.000 |
| ${BEVQTYBOTCAP}_{1}$ | 1,167.284 | 3,471.142 | 0.000 | 18.467 | 136.314 | 522.182 | 25,651.690 |
| ${BEVQTYBOTCAP}_{2}$ | 62,703.960 | 179,157.100 | 1.292 | 1,884.260 | 9,268.390 | 32,787.090 | 1,240,068.000 |
| ${BEVQTYBOTCAP}_{3}$ | 48,207.140 | 164,716.300 | 0.000 | 650.892 | 2,471.715 | 14,519.270 | 1,205,463.000 |

**Table S2. Data correlations of main variables (transformed data).**

|  | | | | | | | | | | |
| --- | --- | --- | --- | --- | --- | --- | --- | --- | --- | --- |
|  | $PET$ | *t* | $NONBEVBOTQTY$ | $BEVQTY$ | ${BOTQTY}_{water}$ | ${BOTQTY}_{juice}$ | ${BOTQTY}_{soda}$ | ${BEVQTYBOTCAP}_{1}$ | ${BEVQTYBOTCAP}_{2}$ | ${BEVQTYBOTCAP}_{3}$ |
|  | | | | | | | | | | |
| $PET$ | 1 | 0.072 | 0.268 | 0.036 | 0.011 | 0.061 | 0.211 | 0.128 | -0.001 | -0.151 |
| *t* | 0.072 | 1 | 0.027 | 0.002 | 0.179 | 0.289 | -0.037 | 0.058 | 0.007 | -0.209 |
| $NONBEVBOTQTY$ | 0.268 | 0.027 | 1 | -0 | 0.382 | 0 | -0 | 0.592 | -0.608 | 0.476 |
| $BEVQTY$ | 0.036 | 0.002 | -0 | 1 | 0 | -0 | 0 | 0 | -0 | 0 |
| ${BOTQTY}_{water}$ | 0.011 | 0.179 | 0.382 | 0 | 1 | -0 | -0 | 0.673 | -0.497 | 0.006 |
| ${BOTQTY}_{juice}$ | 0.061 | 0.289 | 0 | -0 | -0 | 1 | 0 | -0 | 0 | -0 |
| ${BOTQTY}_{soda}$ | 0.211 | -0.037 | -0 | 0 | -0 | 0 | 1 | -0 | 0 | 0 |
| ${BEVQTYBOTCAP}_{1}$ | 0.128 | 0.058 | 0.592 | 0 | 0.673 | -0 | -0 | 1 | -0.482 | 0.298 |
| ${BEVQTYBOTCAP}_{2}$ | -0.001 | 0.007 | -0.608 | -0 | -0.497 | 0 | 0 | -0.482 | 1 | -0.757 |
| ${BEVQTYBOTCAP}_{3}$ | -0.151 | -0.209 | 0.476 | 0 | 0.006 | -0 | 0 | 0.298 | -0.757 | 1 |
|  | | | | | | | | | | |

**Table S3. Feasible Generalized Least Square estimates of additional model specifications of the relationship between product category, bottle capacity, and PET waste collection**. Numbered columns correspond to different model specifications. Numbers in parentheses are standard errors.

|  | | | | | | | |
| --- | --- | --- | --- | --- | --- | --- | --- |
|  | *Dependent variable:* $PET$ | | | | | | |
|  |  | | | | | | |
|  | (1) | (2) | (3) | (4) | (5) | (6) | (7) |
|  | | | | | | | |
| *t* | 74.380 | -56.977 | -62.318 | -48.744 | -65.259 | -107.924 | -385.644^***^ |
|  | (78.074) | (69.603) | (63.066) | (68.777) | (75.911) | (66.552) | (119.389) |
|  |  |  |  |  |  |  |  |
| $NONBEVBOTQTY$ | -0.025 | 0.148 | 0.095 | 0.198^*^ | 0.323^***^ | 0.340^***^ | 2.366^***^ |
|  | (0.036) | (0.097) | (0.100) | (0.101) | (0.122) | (0.124) | (0.538) |
|  |  |  |  |  |  |  |  |
| *AVINCOME* |  |  |  |  |  |  | 33.767 |
|  |  |  |  |  |  |  | (63.958) |
|  |  |  |  |  |  |  |  |
| *AVHHSIZE* |  |  |  |  |  |  | -3,140.779 |
|  |  |  |  |  |  |  | (2,077.538) |
|  |  |  |  |  |  |  |  |
| *UNEMPRATE* |  |  |  |  |  |  | 129.917 |
|  |  |  |  |  |  |  | (147.056) |
|  |  |  |  |  |  |  |  |
| *MEDIANAGE* |  |  |  |  |  |  | -28.085^***^ |
|  |  |  |  |  |  |  | (8.583) |
|  |  |  |  |  |  |  |  |
| ${BOTQTY}_{water}$ |  | 6.510^***^ | 3.118^*^ | 8.007^***^ | 6.728^***^ | -2.922 | -1.865 |
|  |  | (1.372) | (1.587) | (1.634) | (1.409) | (2.616) | (2.411) |
|  |  |  |  |  |  |  |  |
| ${BOTQTY}_{juice}$ |  | 17.386^***^ | 6.160 | 16.243^***^ | 14.630^***^ | -6.032 | 2.627^**^ |
|  |  | (3.032) | (3.944) | (3.004) | (3.449) | (5.753) | (1.049) |
|  |  |  |  |  |  |  |  |
| ${BOTQTY}_{soda}$ |  | -3.322^**^ | -6.685^***^ | -0.612 | -0.829 | -10.475^***^ | -0.277 |
|  |  | (1.273) | (1.485) | (2.040) | (1.845) | (2.643) | (1.854) |
|  |  |  |  |  |  |  |  |
| $BEVQTY$ |  | -9.191^***^ | -4.343^*^ | -13.477^***^ | -9.255^***^ | 10.545^*^ | 0.409 |
|  |  | (2.168) | (2.426) | (3.393) | (2.200) | (5.484) | (0.401) |
|  |  |  |  |  |  |  |  |
| ${BEVQTYBOTCAP}_{1}$ |  |  | 3.107^***^ |  |  | 5.496^***^ | 4.744^***^ |
|  |  |  | (0.709) |  |  | (1.181) | (1.144) |
|  |  |  |  |  |  |  |  |
| ${BEVQTYBOTCAP}_{2}$ |  |  |  | 0.096^*^ |  | -0.254^***^ | -0.142^*^ |
|  |  |  |  | (0.054) |  | (0.082) | (0.082) |
|  |  |  |  |  |  |  |  |
| ${BEVQTYBOTCAP}_{3}$ |  |  |  |  | -0.038^**^ | -0.091^***^ | -0.083^***^ |
|  |  |  |  |  | (0.016) | (0.024) | (0.019) |
|  |  |  |  |  |  |  |  |
|  | | | | | | | |
| R^2^ | 0.076 | 0.137 | 0.138 | 0.167 | 0.251 | 0.315 | 0.32 |
| Adjusted R^2^ | 0.056 | 0.079 | 0.07 | 0.101 | 0.191 | 0.243 | 0.212 |
| Observations | 96 | 96 | 96 | 96 | 96 | 96 | 96 |
|  | | | | | | | |
| *Note:* | ^*^p<0.1; ^**^p<0.05; ^***^p<0.01 | | | | | | |

**Data S1. (Separate file)**

File “Suppl data.xlsx” contains weight, capacity, brand, and category for a sample of bottles of non-alcoholic beverages.
